# Supplementary material for: African Swine Fever: Fast and Furious or Slow and Steady?
Source: Viruses. 2019 Sep 17;11(9):866. doi: 10.3390/v11090866 (PMC6783890; doi:10.3390/v11090866)
Supplement: Supplementary file 1 [file viruses-11-00866-s001.pdf]

viruses

Supplementary Materials

Additional definitions

Katja Schulz*, Franz Josef Conraths, Sandra Blome, Christoph Staubach and Carola Sauter-Louis

Friedrich-Loeffler-Institut, Federal Research Institute for Animal Health, Südufer 10, 17493 Greifswald-Insel Riems, Germany

Franz.Conraths@fli.de
Sandra.Blome@fli.de
Christoph.Staubach@fli.de
Carola.Sauter-Louis@fli.de

*Correspondence:
Katja.Schulz@fli.de; Tel.: +49 38 351 71 803

Morbidity

Morbidity describes the number of diseased animals divided by the number of animals in the population at risk [1]. Rolle and Mayr [2] define morbidity as the number of individuals that are clinically diseased in a certain period of time divided by the individuals at risk present in this period.

The term morbidity can be seen as a generic term of prevalence, which defines the number of diseased individuals in a defined period, and incidence, which describes the number of newly diseased individuals within a specific period of time. However, the terms prevalence and incidence do not necessarily include only clinically ill animals as defined by Rolle and Mayr [2], but may also refer to infected animals with no clinical signs.

Mortality

Mortality, more specifically the cumulative mortality (CM), is defined as the number of deaths that occurred due to a disease during a particular period divided by the number of individuals in the population at the beginning of the defined period [1]. In accordance with Sergeant and Perkins [3], the cause-specific death rate represents the number of deaths in a given time due to the disease of interest, divided by the number of individuals in the total population at risk.

Case fatality ratio

In contrast to mortality, the case fatality ratio depicts the proportion of diseased animals that have died due to the disease [1]. The Case fatality ratio is also described as the number of cases that have died divided by the total number of cases for which the outcome is known [3].

Infectiousness

Infectiousness is a property of the infected host. It defines the period, during which an affected animal is infective, and also the relative amount of the pathogen that can be transmitted by an animal [1].

Infectivity

In contrast to infectiousness, the term infectivity refers to a property of the pathogen. It describes the relative amount of a pathogen needed to initiate infection or the capability to enter the host [4]. The relative amount of a virus that can be transmitted by an animal is often quantified by using the

tissue culture infection dose 50 (TCID50), i.e. the amount of virus required to kill 50% of infected hosts or to produce a cytopathic effect in 50% of inoculated tissue cultures. Thus, the TCID50 can be used to characterize the infectivity of ASFV quantitatively. Infectivity has also been defined as a property of the pathogen, describing its ability to enter the host, settle, proliferate and spread further [2].

Latent period

The latent period is also mainly a property of the pathogen. It describes the period of time that elapses between infection and the shedding of the agent [1].

Contagiousness

The term contagiousness describes the capability of a disease or a pathogen to be transmitted through direct contact [2,3].

Virulence

Virulence represents a property of the pathogen. It describes the ability of 'an infectious agent to cause disease, in a particular host, in terms of severity' [1]. The level of virulence of a pathogen can vary massively, e.g. depending on the virus strain [2]. This holds also true for ASFV [5].

Tenacity

The tenacity of an agent describes its resistance to environmental parameters or, in other words, its capability to remain infectious in a particular setting of extrinsic variables that can affect the agent. This setting may include temperature, humidity, pH and other matrix effects, caused by factors in the surroundings of the agent, e.g. blood, tissue, soil or water. The tenacity of an infectious agent can for example be quantified by describing its temperature- or pH-stability [2].

References

1. Thrusfield, M. *Veterinary Epidemiology*; John Wiley & Sons Ltd: Edinburgh, 2018; Vol. 4.
2. Rolle, M.; Mayr, A. *Medizinische Mikrobiologie, Infektions- und Seuchenlehre*; Enke Verlag: Stuttgart, 2007.
3. Sergeant, E.; Perkins, N. *Epidemiology for Field Veterinarians*; CABI: Croydon, 2015.
4. Rodriguez-Lazaro, D.; Cook, N.; Ruggeri, F.M.; Sellwood, J.; Nasser, A.; Nascimento, M.S.; D'Agostino, M.; Santos, R.; Saiz, J.C.; Rzezutka, A., et al. Virus hazards from food, water and other contaminated environments. *FEMS Microbiol. Rev.* **2012**, *36*, 786-814, doi:10.1111/j.1574-6976.2011.00306.x.
5. Blome, S.; Gabriel, C.; Beer, M. Pathogenesis of African swine fever in domestic pigs and European wild boar. *Virus Res.* **2013**, *173*, 122-130, doi:10.1016/j.virusres.2012.10.026.

© 2019 by the authors. Submitted for possible open access publication under the terms and conditions of the Creative Commons Attribution (CC BY) license (http://creativecommons.org/licenses/by/4.0/).
